# Tetraspanin Scaffold Proteins Function as Key Regulators of Hematopoietic Stem Cells

**DOI:** 10.3389/fcell.2020.00598

**Published:** 2020-07-09

**Authors:** Victoria D. Balise, Chelsea A. Saito-Reis, Jennifer M. Gillette

**Affiliations:** ^1^Department of Pathology, University of New Mexico Health Sciences Center, Albuquerque, NM, United States; ^2^Comprehensive Cancer Center, The University of New Mexico, Albuquerque, NM, United States

**Keywords:** tetraspanin, hematopoietic stem cell, bone marrow niche, quiescence, adhesion, migration

## Abstract

Hematopoietic stem and progenitor cells (HSPCs) are responsible for the development, maintenance, and regeneration of all the blood forming cells in the body, and as such, are critical for a number of patient therapies. For successful HSPC transplantation, stem cells must traffic through the blood and home to the bone marrow (BM) microenvironment or “niche,” which is composed of soluble factors, matrix proteins, and supportive cells. HSPC adhesion to, and signaling with, cellular and extracellular components of the niche provide instructional cues to balance stem cell self-renewal and differentiation. In this review, we will explore the regulation of these stem cell properties with a focus on the tetraspanin family of membrane proteins. Tetraspanins are molecular scaffolds that uniquely function to distribute proteins into highly organized microdomains comprising adhesion, signaling, and adaptor proteins. As such, tetraspanins contribute to many aspects of cell physiology as mediators of cell adhesion, trafficking, and signaling. We will summarize the many reports that identify tetraspanins as markers of specific HSPC populations. Moreover, we will discuss the various studies establishing the functional importance of tetraspanins in the regulation of essential HSPC processes including quiescence, migration, and niche adhesion. When taken together, studies outlined in this review suggest that several tetraspanins may serve as potential targets to modulate HSPC interactions with the BM niche, ultimately impacting future HSPC therapies.

## Introduction

Hematopoietic stem and progenitor cells (HSPCs) are responsible for the lifelong production of all mature blood and immune cells. As such, HSPCs are critical not only for maintaining homeostasis of the hematopoietic system, but also for responding to stresses such as infection, chemotherapy, or malignant hematopoiesis. The dynamic regulation of the hematopoietic system is not maintained by HSPCs alone, but rather is coordinated with essential support from the surrounding microenvironment or niche (Dexter et al., 1977; Schofield, 1978). HSPCs primarily reside in the bone marrow (BM), which is a complex microenvironment consisting of various cellular and extracellular components all with the capacity to regulate HSPC function and maintenance (Zhang et al., 2003; Morrison and Scadden, 2014). More specifically, there is dynamic interplay between the physical interactions of HSPCs and niche compartments that respond to physiological cues. For example, both circadian oscillations and stress impact the release and trafficking of HSPCs from the BM (Mendez-Ferrer et al., 2009), which leads to their circulation and when necessary activation (Huang et al., 2007), or their return to the BM in the process of homing (Lapidot et al., 2005). Clinically, the natural egress and homing of HSPCs are harnessed to collect and successfully transplant HSPCs for treatment of diseases such as hematologic malignancies and BM failure (Hatzimichael and Tuthill, 2010).

Emerging experimental evidence highlights the impact of tetraspanins on the regulation of HSPC activation, HSPC/niche interactions, and the dynamic trafficking of HSPCs into and out of the BM. Thus, in this mini-review, we feature the contributions of specific tetraspanin family members that have been identified as critical modulators of HSPC function.

## Tetraspanins

**FIGURE 1:**
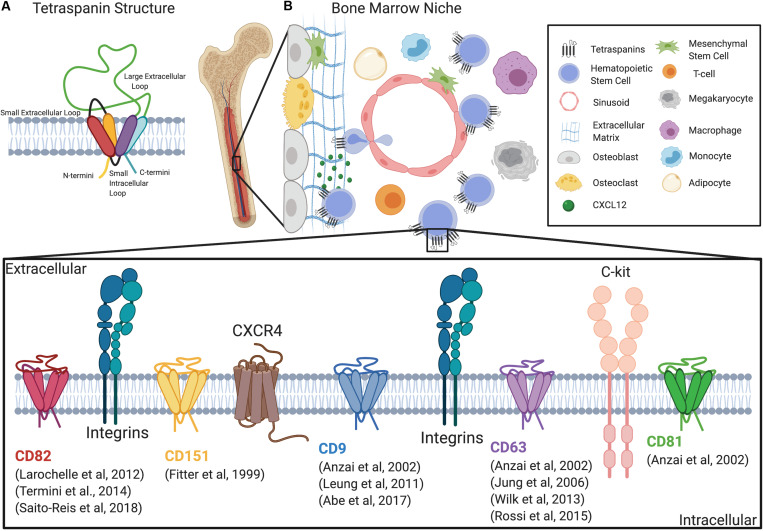
Tetraspanins regulate HSC adhesion, migration, and signaling within the niche. **(A)** Schematic of the tetraspanin molecular structure. **(B)** Diagram depicting the bone marrow niche and the cell types that regulate HSC function including mesenchymal stem cells, endothelial cells, megakaryocytes, macrophages, osteoblasts, osteoclasts, and adipocytes. Additionally, a magnified diagram of the HSPC membrane depicts different tetraspanins involved in adhesion, migration, and signaling, as well as known interacting molecules.

The tetraspanin family of membrane scaffold proteins are expressed in all multicellular eukaryotes, with 33 known tetraspanins in humans (Todres et al., 2000; Boucheix and Rubinstein, 2001; Adell et al., 2004). Tetraspanins are characterized by four transmembrane domains, two extracellular loops: one small extracellular loop (SEL or EC1) and one larger extracellular loop (LEL or EC2), a short intracellular loop, and two short intracellular tails (Stipp et al., 2003; Hemler, 2005) (Figure 1A). Tetraspanins are defined by conserved amino acid sequences within EC2 consisting of four or more cysteine residues and a highly conserved CCG motif (Hemler, 2003; Stipp et al., 2003). Additionally, analysis of alternative splice sites within the tetraspanin family recently identified a significant number of novel tetraspanin isoforms with large structural variability (Hochheimer et al., 2019). Tetraspanins protrude only 4–5 nm above the transmembrane and thus can be overlooked by biochemical and immunological detection (Hemler, 2005). Recent crystal structure studies have provided significant insight including the identification of a cholesterol-binding pocket created by the four transmembrane domains of tetraspanin CD81 (Zimmerman et al., 2016). In fact, cholesterol-binding motifs have now been identified in 30 of the 33 human tetraspanins (Huang et al., 2020). Moreover, the crystal structure for human CD9 describes how its reversed cone-shaped structure generates membrane curvature in a crystalline lipid layer, which also likely explains the localization of CD9 to regions of high membrane curvature (Umeda et al., 2020).

Tetraspanins function as membrane scaffolds through the formation of tetraspanin-enriched microdomains (TEMs), which compartmentalize membrane adhesion and signaling receptors as well as intracellular signaling molecules (Termini and Gillette, 2017; van Deventer et al., 2017). Tetraspanins are also subject to post-translational modifications that include, but are not limited to, palmitoylation and *N*-glycosylation (Termini and Gillette, 2017). For example, tetraspanins CD9, CD37, CD53, CD63, CD81, CD82, and CD151 were found to incorporate palmitate to membrane proximal cysteine residues, which facilitates the stability of tetraspanins within the membrane and promotes formation of TEMs (Charrin et al., 2002; Levy and Shoham, 2005). Additionally, many tetraspanins are glycosylated, which can contribute to their structure and heterogeneity in size (Yunta and Lazo, 2003) and it is also important for regulating protein–protein interactions (Stipp et al., 2003). While some tetraspanins such as CD9, CD81, and CD82 are ubiquitously expressed (Maecker et al., 1997), other tetraspanins including CD37, CD53, and Tssc6/tspan32 are restricted to hematopoietic cells (Schwartz-Albiez et al., 1988; Maecker et al., 1997). Therefore, the differential expression of tetraspanins in various tissues may translate into specific functions within different cell types. Tetraspanins are also one the most commonly found protein classes in extracellular vesicles (EVs) (Andreu and Yanez-Mo, 2014). In fact, EVs can be categorized based on the enrichment of tetraspanins (Kowal et al., 2016). Collectively, tetraspanins, and the TEMs they assemble, act as molecular facilitators that spatiotemporally organize membrane-associated proteins that drive diverse cellular processes, including those critical for HSPC function, such as quiescence, self-renewal, differentiation, adhesion, migration, and signaling (Table 1 and Figure 1B).

**TABLE 1:** Tetraspanins and HSC Regulation.

Tetraspanin	Cell type	Function	References

**Tetraspanins identified as surface markers**			
CD9	Murine multipotent hematopoietic progenitor cell line	Marker for HPCs	Bruno et al., 2004
	Murine HSPCs	Marker on murine LT-HSC, short-term HSC, and multipotent progenitor cell populations	Forsberg et al., 2005
	Murine HSPCs	Marker for murine HSCs	Karlsson et al., 2013
	Human cord blood	Cord blood stem cell maker	Zhao et al., 2006
	Porcine hematopoietic progenitors	Negative enrichment marker for HPCs	Heinz et al., 2002
CD82	Human cord blood and human peripheral blood	Identification of tetraspanin on hematopoietic progenitor cells	Burchert et al., 1999
	Murine HSCs and MPP	Proteomic analysis identified high CD82 expression in HSCs in comparison to MPP	Cabezas-Wallscheid et al., 2014
	Murine HSCs and HSPCs	Gene expression analysis demonstrated high CD82 expression in the LT-HSC compared to ST-HSC and MPP populations	Hur et al., 2016
CD53	Murine hematopoietic cell lineages	Differential expression of CD53 within the HSPC population	Guo et al., 2013
CD81	Human CD34^+^ cord blood HSPCs	Marker for differentiation of lymphohematopoietic stem and progenitor cells	Ma et al., 2001

**Tetraspanins identified to regulate quiescence**			

CD81	Murine HSCs	Regulation of re-entry of HSC quiescence	Lin et al., 2011
CD82	Human peripheral blood HSPCs	Mediation of HSPC interaction with osteoblasts and HSPC quiescence	Larochelle et al., 2012
	Murine HSCs and HSPCs	Regulation of LT-HSC quiescence	Hur et al., 2016
	Murine HSPCs	Regulation of LT-HSC quiescence	Saito-Reis et al., 2018

**Tetraspanins identified to regulate asymmetric cell division and differentiation**			

CD63	Human umbilical cord blood, bone marrow, and peripheral blood	Marker for asymmetric HSC division	Beckmann et al., 2007; Giebel and Beckmann, 2007
CD53	Human umbilical cord blood, bone marrow, and peripheral blood	Marker for asymmetric HSC division	Beckmann et al., 2007; Giebel and Beckmann, 2007
	Hematopoietic cell line, BaF/3	B-cell development	Mansson et al., 2007
	Murine bone marrow	Early B-Cell development by regulating IL-7R	Greenberg et al., 2020
CD81	Human thymocytes	B lymphocyte development	Tedder et al., 1994
	Human blood samples	Decreased memory B cell numbers in CD81-deficient patients	van Zelm et al., 2010
CD9	Human bone marrow CD34^+^	Stimulates differentiation to megakaryocytic lineages	Clay et al., 2001
	Human cord blood CD34^+^	Dendritic cell marker	Caux et al., 1996
	Murine megakaryocyte-erythroid progenitors	High expression of CD9 promotes self- renewal	Weiss-Gayet et al., 2016
	Pluripotent hematopoietic cell line, EML-C1	Regulation of HSC differentiation, proliferation, and self-renewal through stromal cell expression	Aoyama et al., 1999; Oritani et al., 2000

*HPC, hematopoietic progenitor cell; HSPC, hematopoietic stem and progenitor cell; MPP, multipotent progenitor; LT-HSC, long-term hematopoietic stem cell; ST-HSC, short-term hematopoietic stem cell.*

## Tetraspanins: Hematopoietic Stem Cell (HSC) Surface Markers

The identification and stratification of HSCs and their progeny is largely based on the use of cell surface markers. For example, long-term hematopoietic stem cells (LT-HSCs), the most primitive of hematopoietic cells with the ability to self-renew and repopulate the entire blood and immune system, are characterized not by a single surface marker, but rather a combination of surface markers. Several studies have also identified specific tetraspanins as surface markers that can enrich various HSPC populations.

The tetraspanin CD9 was identified as a positive marker for HSCs and HSPCs in both murine and human models. Initially, Bruno et al. discovered high CD9 gene expression within a murine multipotent hematopoietic progenitor cell (HPC) line, but subsequent studies identified CD9 on murine LT-HSC, short-term HSC (ST-HSC), and multipotent progenitor (MPP) cell populations (Bruno et al., 2004; Forsberg et al., 2005). Additionally, CD9^*high*^ cell populations were identified to not only have HSC phenotypes, but also to be functional in murine transplants (Karlsson et al., 2013). CD9 has also been used as a positive marker to identify human umbilical cord blood stem cells (Zhao et al., 2006). In contrast, a porcine study screening antibodies to identify markers for negative enrichment of HPCs, discovered seven markers, including CD9 (Heinz et al., 2002). Here, HPCs with negative or low expression of CD9 fell within the side population, suggesting that discrepancies may exist between different species with respect to HSPC markers.

Tetraspanin CD82 (KAI1) is also described to be abundantly expressed on the surface of primitive and committed HSPCs isolated from peripheral blood (Burchert et al., 1999). Interestingly, in this study, the level of CD82 expression decreased upon differentiation of CD34^+^ HSPCs, but was found to increase in leukemias such as CML, AML, and CLL. More recent proteomic analysis of mouse HSCs and MPPs also identified high CD82 expression in HSCs, when compared to MPPs (Cabezas-Wallscheid et al., 2014). Similarly, gene expression analysis demonstrated that CD82 was expressed predominantly in the LT-HSC rather than the ST-HSC and MPP populations (Hur et al., 2016). In contrast, this study found that other members of the tetraspanin superfamily (CD9, CD37, CD81, and CD151) were expressed in every HSPC population at the mRNA level. However, Guo et al. (2013) identified CD53 to be differentially expressed within the murine HSPC population by single-cell gene expression analysis. Additionally, CD81 was described as a marker for the development of lymphohematopoietic stem and progenitor cells (Ma et al., 2001) and the expression of Tssc6 has been confirmed on HPCs and various HSC lines (Robb et al., 2001). While tetraspanin expression appears to vary across HSPC populations, it is clear that certain tetraspanins can be used to enrich specific hematopoietic cell fractions, which may improve stem cell therapies.

## Tetraspanins: HSC Quiescence

Within the BM microenvironment, HSCs primarily reside in a quiescent state (Wilson et al., 2008). However, upon injury, HSCs are activated into cycle, but then must ultimately return to quiescence (Wilson et al., 2008). Therefore, HSC quiescence is not only important for protecting the stem cell pool from mutations accumulated via active cycling, but also for sustaining the HSC pool over the lifespan of an organism (Li, 2011). Tetraspanins CD81 and CD82 have both been described as important modulators of HSC quiescence.

The spatial distribution of CD81 on the surface of murine HSCs was shown to be important for the re-entry of HSCs into quiescence from a highly proliferative state (Lin et al., 2011). More specifically, the polarization of CD81 was shown to promote the deactivation of Akt signaling and the nuclear translocation of FoxO1a, leading to HSC quiescence. Similarly, work from our group identified a change in the distribution of CD82 as human CD34^+^ cells progress through the cell cycle (Larochelle et al., 2012). Quiescent, G0 cells were found to have a polarized organization of CD82, which was redistributed throughout the plasma membrane upon cell cycle entry. Together, these data highlight the critical contribution of tetraspanin membrane organization to the signal transduction driving quiescence. In both studies, the tetraspanin membrane organization was described at the micron scale. Therefore, future studies will be required to determine how tetraspanin assembly into nanoscale TEMs and the dynamic modulation of TEMs impacts quiescence signaling of HSCs.

A number of tetraspanin knock out (KO) mice have been generated, but up until now, only two have displayed an HSC defect. CD82KO animals exhibit a reduction in LT-HSCs, resulting from increased stem cell activation and reduced quiescence signaling (Hur et al., 2016; Saito-Reis et al., 2018). In contrast, HSCs from CD81KO mice appear to proliferate similarly to control animals, but demonstrate a reduced ability to reenter quiescence after stimulation (Lin et al., 2011). Interestingly, the signaling molecules described to be involved in the regulation of quiescence include both Akt and TGFβ signaling (Lin et al., 2011; Hur et al., 2016). The modulation of Akt and TGFβ signaling by CD81 and CD82, respectively, likely involves the scaffolding property of these tetraspanins and their ability to cluster receptors and downstream signaling molecules into TEMs. Future experiments utilizing advanced imaging technologies will be required to confirm how tetraspanins and the dynamic assembly of TEMs influence these quiescence signaling pathways.

## Tetraspanins: Asymmetric Division and Differentiation

The balance between HSC self-renewal and differentiation is thought to be regulated by asymmetric cell division, where a cell produces a daughter cell that retains intrinsic stem cell properties plus one that initiates differentiation (Ho, 2005; Caocci et al., 2017). Previous work identified a series of molecules expressed on HSCs that undergo asymmetric division, including CD133, CD71, CD62L, CD34, and the tetraspanins, CD53 and CD63 (Beckmann et al., 2007; Giebel and Beckmann, 2007). In fact, CD53, in combination with CD63 served as more stringent markers for asymmetric division than the previously described CD133 and CD34 expression profiles (Beckmann et al., 2007; Giebel and Beckmann, 2007). In this study, both CD53 and CD63 were linked to the endosomal compartment, which plays a critical role in protein trafficking (Beckmann et al., 2007). Moreover, it has been suggested that the asymmetric segregation of endosomes might provide a more general and evolutionary conserved mechanism for asymmetric cell division (Murke et al., 2015).

Specific tetraspanin expression has also been linked to differentiation of hematopoietic cells. For example, CD53 was shown to be a genetic target for Early B-cell Factor-1, a critical transcription factor for B-cell development (Mansson et al., 2007). More recently, an increase in CD53 expression was demonstrated in early B-cell development where a physical interaction between CD53 and the Interleukin-7 receptor modulates key signaling in early B cell differentiation (Greenberg et al., 2020).

Also critical for B lymphocyte development and humoral immunity is the CD19–CD21–CD81 complex (Tedder et al., 1994), where tetraspanin CD81 associates with CD19 in the endoplasmic reticulum and regulates CD19 surface transport. In fact, CD81-deficient patients were characterized by decreased memory B cell numbers and an absence of CD19 surface expression, which further demonstrates an essential, non-redundant role of CD81 (van Zelm et al., 2010). Biochemical analysis of the CD19–CD21–CD81 complex also identified an association with tetraspanins CD82 and CD9 (Horvath et al., 1998). A comprehensive description of tetraspanin activity in B cell development can be found in the following review (Zou et al., 2018).

Tetraspanin CD9 is also a regulator of HSPC differentiation with specific roles in the differentiation of the megakaryocytic, B-lymphoid, and myeloid lineages (Brosseau et al., 2018). For instance, high CD9 expression on CD34^+^ cells promotes differentiation to megakaryocytic lineages (Clay et al., 2001). Additionally, CD9 surface expression was identified to characterize a population of dendritic cells that were differentiated *in vitro* from CD34^+^ HPCs isolated from human cord blood (Caux et al., 1996). Moreover, a subset of CD9^*High*^ committed megakaryocytic progenitors were shown to exhibit self-renewal and lineage plasticity downstream of Notch stimulation (Weiss-Gayet et al., 2016). Further study of CD9 revealed its expression on a large variety of other hematopoietic cells, including platelets, T lymphocytes, mast cells, eosinophils, and basophils (Fernvik et al., 1995; Tai et al., 1996) as well as critical BM stromal cells that can also regulate HSPC activity (Aoyama et al., 1999; Oritani et al., 2000). For example, when the pluripotent hematopoietic cell line, EML-C1, was cultured on stromal cells treated with an anti-CD9 antibody, a block in HSC differentiation, proliferation, and self-renewal was measured (Aoyama et al., 1999). Interestingly, CD9 and CD63 were also identified to be enriched in EVs produced by stromal cell lines that can differentially support HSPCs (Stik et al., 2017). It has been suggested that tetraspanins within EVs may facilitate target cell binding and uptake (Morelli et al., 2004); however, whether tetraspanins serve this role in HSC/niche communication requires further study. At this time, it is clear that tetraspanins play a key role in the regulation of niche interactions, which will be further discussed below.

## Tetraspanins: HSC Adhesion, Migration, and Signaling Within the Niche

The BM niche is a particularly complex microenvironment that consists of a diverse cellular repertoire critical for regulating HSC function and maintenance. Currently, it is believed that HSCs are predominantly localized to the perivascular region of the BM, with endothelial cells and mesenchymal stromal cells serving to secrete factors that promote HSC maintenance (Crane et al., 2017). However, other cell types also directly or indirectly regulate HSC niches, including megakaryocytes, macrophages, osteoblasts, osteoclasts, adipocytes, and other stromal cell populations (Morrison and Scadden, 2014). Additionally, the extracellular matrix (ECM) within the BM acts as a supportive tissue for the maintenance of HSCs (Discher et al., 2009). The different components of the BM microenvironment are depicted in Figure 1B and have been extensively reviewed (Crane et al., 2017; Szade et al., 2018; Pinho and Frenette, 2019).

The dynamic adhesion and migration behaviors of HSCs within the BM are significantly regulated by multiple adhesion proteins, including the integrin family of adhesion molecules (Klamer and Voermans, 2014). Integrin complexes, α4β1 and αLβ2 play an important role in HSC adhesion to the vasculature to aide in trans-endothelial migration (Peled et al., 2000). Additionally, integrin complexes α4β1 and α6β1, are both important for homing of HSCs to the BM. Studies using antibodies to block either α4 or α6 resulted in an inhibition of BM homing of HSPCs (Papayannopoulou et al., 1995; Qian et al., 2006). Moreover, in combination, α4 and α6 antibodies synergistically inhibited homing of ST-HSCs (Qian et al., 2006). Interestingly, an increase in homing of progenitors to spleen was only detected with antibodies against α4 (Papayannopoulou et al., 1995), suggesting distinct roles of integrins in HSCs homing. Both α4β1 and α5β1 were also found to mediate chemotaxis of CD34^+^ HPCs on fibronectin, while α4β1 alone mediates adhesion (Carstanjen et al., 2005). Integrin interactions with tetraspanins have been well characterized (Berditchevski, 2001). In fact, tetraspanins have been described to impact integrin expression, signaling, and compartmentalization. Thus, much of the contribution of tetraspanins to the regulation of HSC adhesion and migration within the niche involves integrin-mediated interactions (Figure 1B).

Our work identified a critical role for the tetraspanin CD82 in mediating HSPC/niche adhesion. CD82 was found to be enriched at the site of HSPC contact with osteoblasts and pre-treatment of CD34^+^ cells with an anti-CD82 monoclonal antibody resulted in reduced adhesion, homing, and engraftment (Larochelle et al., 2012). Follow-up studies with a human hematopoietic cell line demonstrated that CD82 promotes fibronectin adhesion through the regulation of integrin α4β1 organization (Termini et al., 2014). Super-resolution imaging studies identified CD82 as a modulator of integrin density, which contributed to changes in ECM adhesion. Similarly, a previous study focused on CD151 identified interactions with integrins β1 and αIIbβ3 as critical for promoting HSPC adhesion to various ECMs (Fitter et al., 1999) and both CD81 and CD82 were shown to mediate α4β1 adhesion of erythroblasts to Vascular Cell Adhesion molecule-1 (Spring et al., 2013). More recently, our work using the CD82KO mouse model identified a homing and engraftment defect of HSPCs, which was linked to altered Rac1 activity (Saito-Reis et al., 2018), further illustrating that CD82 may serve as a therapeutic target to modulate HSPC adhesion and migration.

Tetraspanin CD9 has also been described as a regulator of HSPC adhesion and homing. Pretreatment of cord blood CD34^+^ cells with an anti-CD9 monoclonal antibody inhibited CXCL12-mediated transendothelial migration; however, adhesion to fibronectin and endothelial cells was enhanced (Leung et al., 2011). Additionally, antibody pretreatment of CD34^+^ cells significantly impaired their homing to the BM and sorted CD34^+^ CD9^–^ cells displayed lower BM homing capacity compared with that of total CD34^+^ cells. More recently, a separate group characterized human CD34^–^ HSCs isolated from cord blood, finding that engraftment in mice and sheep was limited due to a decrease in CD9 expression and an increase in the inhibitory homing molecule, CD26 (Abe et al., 2017). Collectively, these data demonstrate that CD9 expression also contributes to HSC migration and niche adhesion, although identification of its mechanistic role and interacting partners remains unclear. Studies evaluating mature hematopoietic cells have identified a role for CD9 in regulating cell adhesion-mediated by integrins, αLβ2 and α5β1 (Reyes et al., 2015, 2018; Machado-Pineda et al., 2018). Thus, future studies focused on the role of CD9 and HSCs are likely to uncover key integrin interactions.

In addition to its enrichment in HSC endosomes, tetraspanin CD63 was found to interact with the tissue inhibitor of metalloproteinase-1 (TIMP1), a protein important for HSC quiescence and long-term engraftment (Jung et al., 2006). TIMP1 was shown to bind to the CD63/β1 integrin complex on the surface of human CD34^+^ HSPCs to induce adhesion and migration (Wilk et al., 2013). Moreover, this group determined that homing and short-term engraftment of HSPCs were also increased upon exogenous stimulation with TIMP1. The interaction of TIMP1 and CD63 was also shown to impact HSPC proliferation through the activation of the PI3K/AKT signaling pathway (Rossi et al., 2015) and the enrichment of myeloid progenitors, impacting granulopoiesis (Kobuch et al., 2015). Additionally, HSC proliferation, self-renewal, and maintenance of niche interactions are regulated by the tyrosine kinase receptor c-kit (CD117) expressed on the surface of HSPCs and its ligand stem cell factor (Barker, 1997; Kent et al., 2008). Using a combination of immunoprecipitation and co-localization experiments, tetraspanins CD9, CD63, and CD81 were shown to interact with c-kit (Anzai et al., 2002). Functionally, this study suggested that tetraspanins negatively modulate c-kit signaling and thus may regulate receptor sensitivity to ligand within the BM niche.

## Conclusion

Tetraspanins are a family of proteins that regulate multiple cellular processes through their organization of membrane-associated proteins into TEMs. This review explores the tetraspanins currently known to modulate various HSPC functions. Our description of the diverse HSPC processes regulated by several tetraspanins highlights the need to further investigate the mechanistic role for tetraspanins in the regulation of HSC signaling and niche interactions. Future studies focused on how tetraspanins dynamically modulate the compartmentalization of critical HSPC signaling and adhesion molecules will help us understand the specific mechanisms used by this family of proteins to control unique HSPC activities. Now that sophisticated imaging techniques are becoming more widely available, we are likely to learn more about how tetraspanins contribute to the formation and stabilization of signaling and adhesion complexes essential for HSC function. As several tetraspanin family members are enriched in HSPCs, it will also be critical to investigate how tetraspanins may work in concert to modulate interacting protein partners and downstream signaling that contribute to HSPC/niche interactions. Finally, the generation and analysis of HSC-specific tetraspanin KO mice will be essential for separating the critical roles for tetraspanins specifically within HSCs from those required in the BM niche. Collectively, an advanced understanding of how tetraspanins contribute to HSPC function may lead to future breakthroughs in the isolation and the therapeutic use of HSPCs.

## Author Contributions

VB, CS-R, and JG contributed to manuscript writing. VB generated the figures. All authors approved the final version of the manuscript.

## Conflict of Interest

The authors declare that the research was conducted in the absence of any commercial or financial relationships that could be construed as a potential conflict of interest.
